# Supplementary figures and images for: Transfer of the synechan biosynthesis and regulatory pathway enables sulfated polysaccharide production in Synechococcus elongatus PCC 7942
Source: Sci Rep. 2026 Apr 28;16:13012. doi: 10.1038/s41598-026-46439-4 (PMC13125312; doi:10.1038/s41598-026-46439-4)

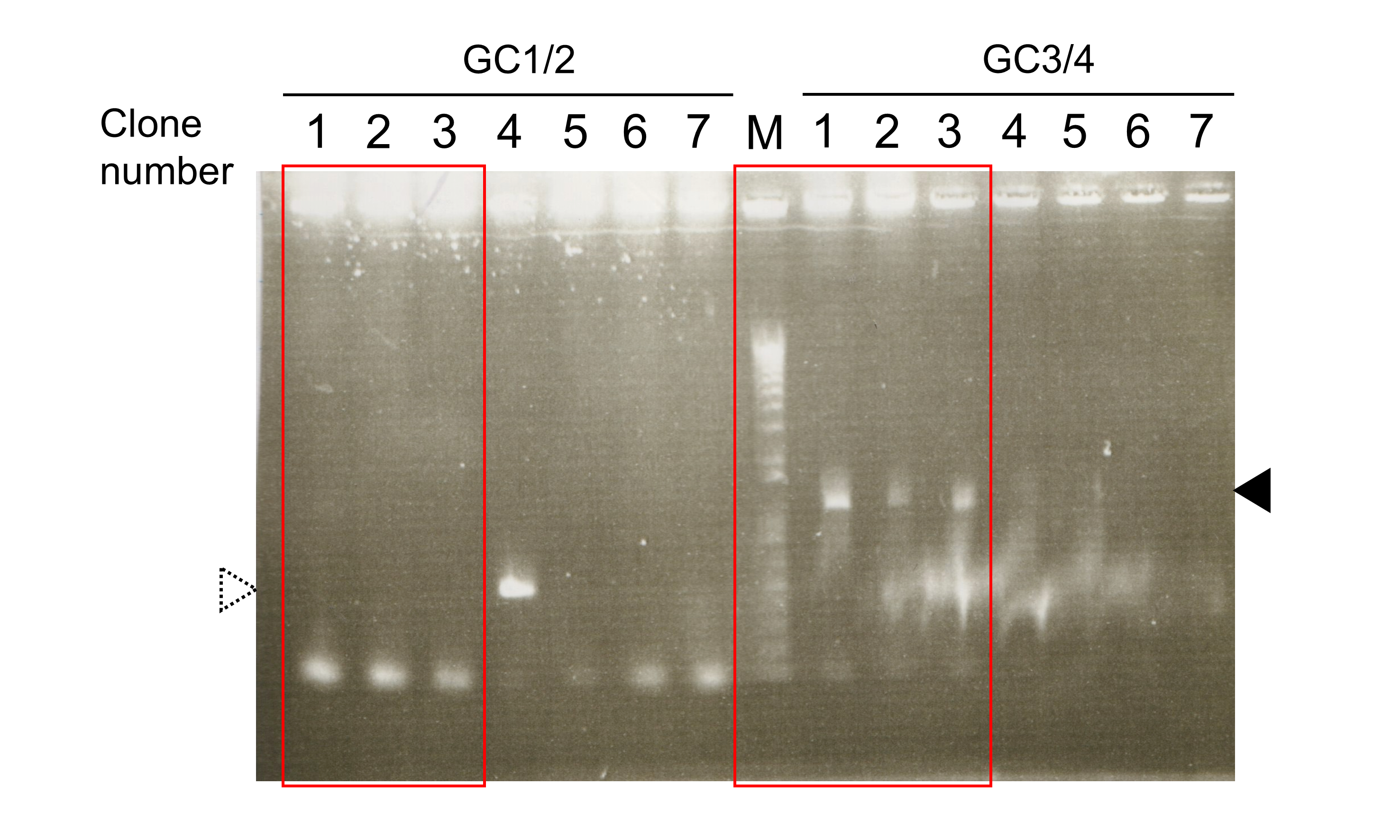

Supplement: Supplementary file 4 — Supplementary Data S3. [file 41598_2026_46439_MOESM4_ESM.tif]

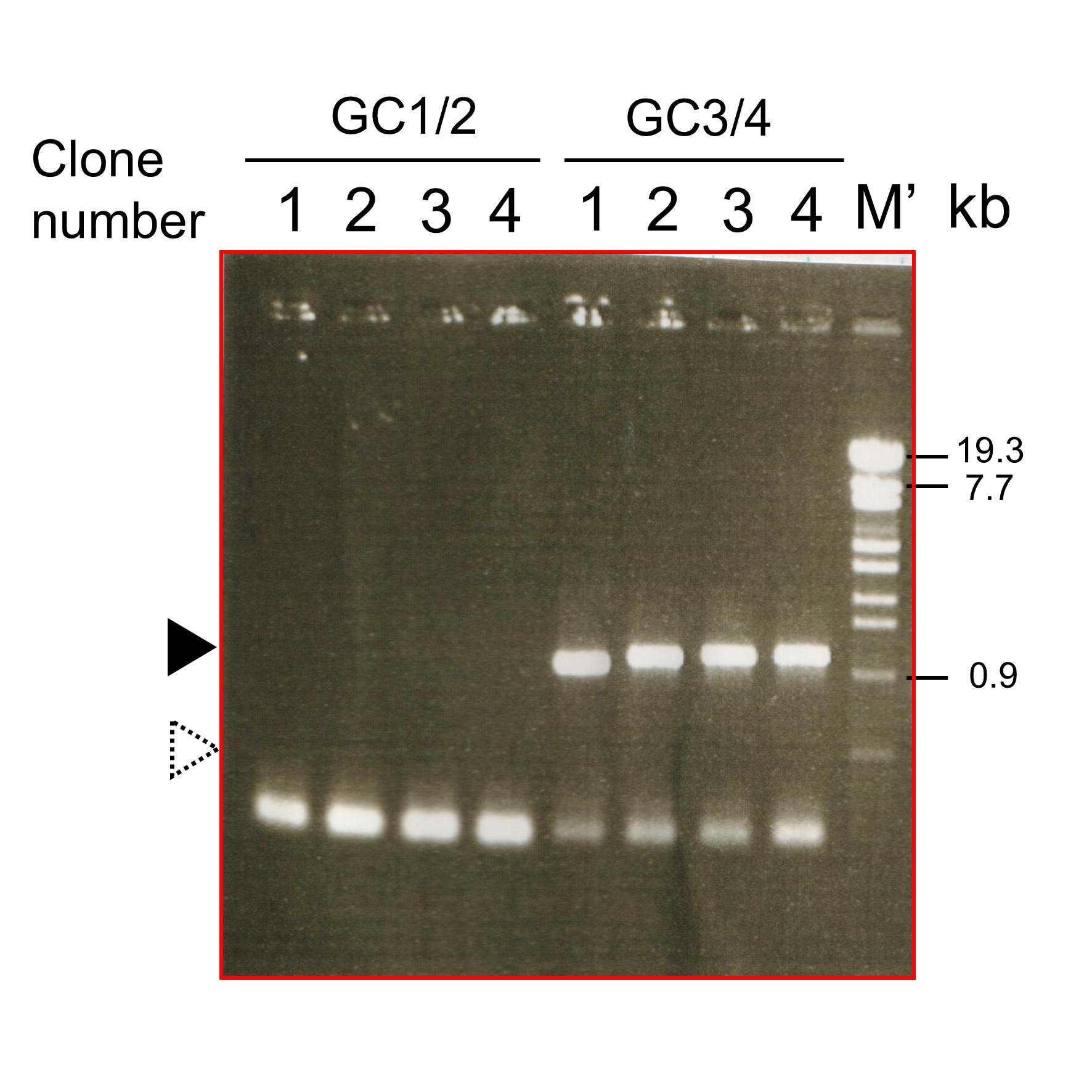

Supplement: Supplementary file 5 — Supplementary Data S4. [file 41598_2026_46439_MOESM5_ESM.tif]

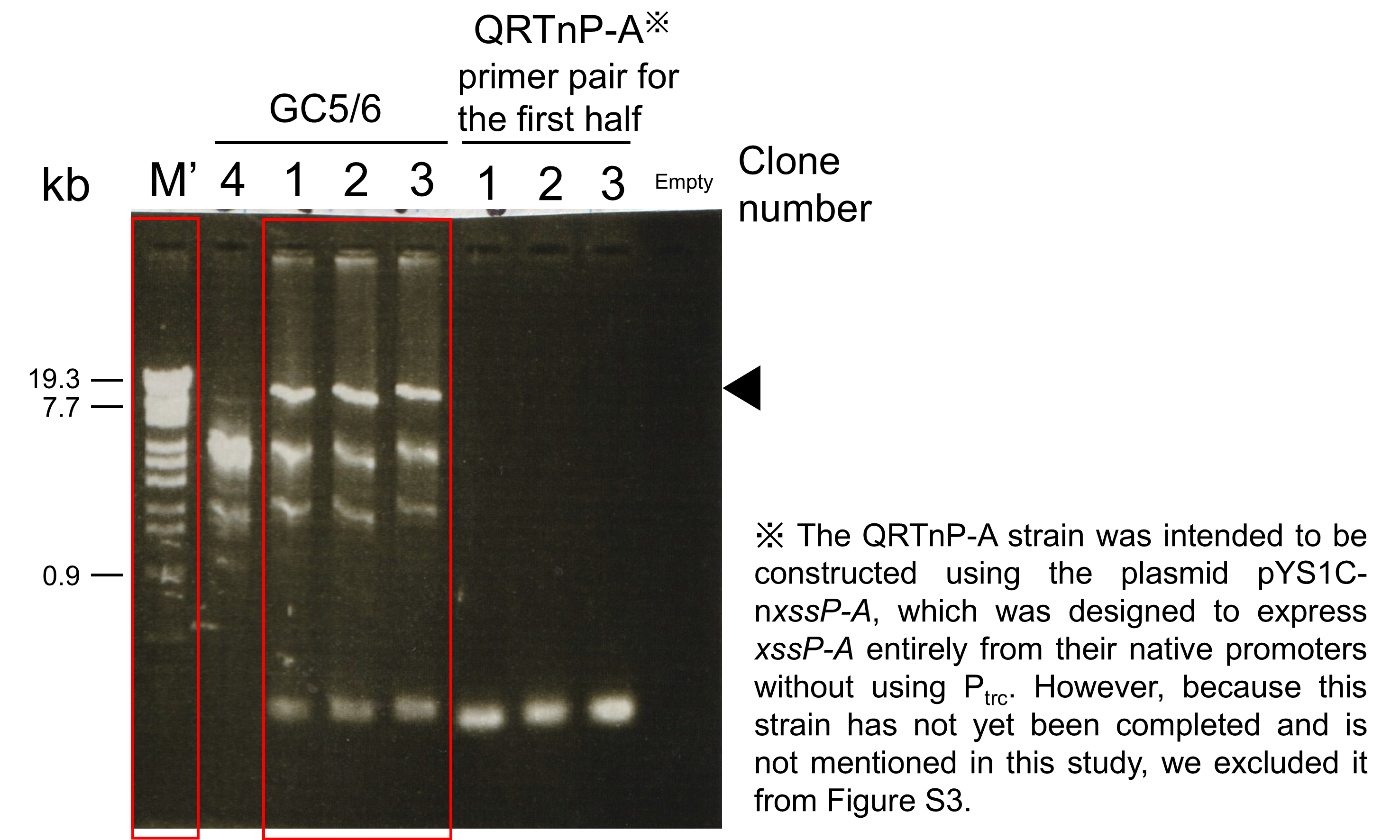

Supplement: Supplementary file 6 — Supplementary Data S5. [file 41598_2026_46439_MOESM6_ESM.tif]

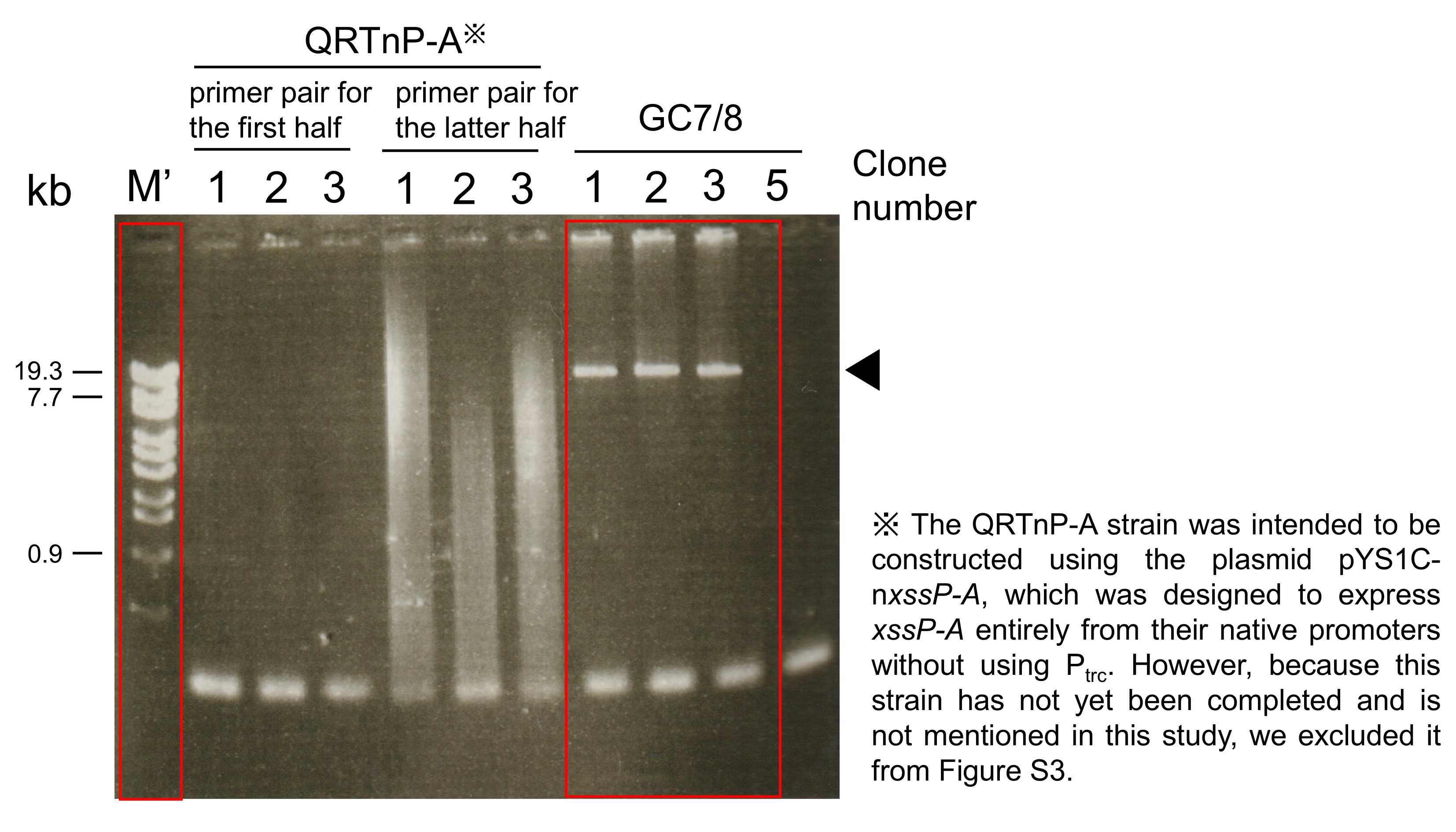

Supplement: Supplementary file 7 — Supplementary Data S6. [file 41598_2026_46439_MOESM7_ESM.tif]
